# Supplementary material for: Revealing topology in metals using experimental protocols inspired by K-theory
Source: Nat Commun. 2023 May 27;14:3071. doi: 10.1038/s41467-023-38862-2 (PMC10224923; doi:10.1038/s41467-023-38862-2)
Supplement: Supplementary file 1 — Supplementary Information [file 41467_2023_38862_MOESM1_ESM.pdf]

Supplementary Information for Revealing Topology in Metals using Experimental Protocols Inspired by K-Theory

Wenting Cheng,[1,*] Alexander Cerjan,[2,†] Ssu-Ying Chen,[1,‡]
Emil Prodan,[3,§] Terry A. Loring,[4,¶] and Camelia Prodan[1,**]

[1]*Department of Physics, New Jersey Institute of Technology, Newark, NJ, USA*
[2]*Center for Integrated Nanotechnologies, Sandia National Laboratories, Albuquerque, New Mexico 87185, USA*
[3]*Department of Physics, Yeshiva University, New York, NY, USA*
[4]*Department of Mathematics and Statistics, University of New Mexico, Albuquerque, New Mexico 87131, USA*

SUPPLEMENTARY NOTE 1: SYMMETRY VALIDATION

To demonstrate that the chiral symmetry is present for our acoustic crystals, we first focus on the insulating component of the system considered in the main text, which was claimed to supply an accurate realization of the SSH model. The physical model itself is shown in Supplementary Figure 1**d**. Note that the acoustic chain was terminated such that both ends are topologically non-trivial.

We start by reporting the COMSOL-simulated resonant spectra of SSH acoustic crystals generated with different geometries of the grooved channels. As one can see in Supplementary Figure 1**a**, the spectrum is ungapped for a uniform channel, but a bulk spectral gap opens as soon as the connecting channels are set in an alternating geometry. Furthermore, with a good degree, the bulk spectrum remains symmetric relative to the middle of the bulk spectral gap (the small deviations are less than 5% when compared with the overall width of the bulk spectrum). In the same panel, one can observe the expected edge resonant modes, whose energies are pinned with a high degree in the middle of the bulk spectral gap. For our final experimental design, we chose the geometry highlighted in red in Supplementary Figure 1**a**, for which the COMSOL-simulated spectrum display an exact chiral symmetry.

The above features, are semi-quantitatively reproduced experimentally, as reported in panels **b** and **c** of Supplementary Figure 1. For these measurements, the speaker and the microphone were inserted in the same resonator via two holes open at the top and the speaker's frequency was swept from 3.5 kHz to 5.5 kHz. The measurements were repeated for all resonators and the collected data was assembled in the local density of states plot shown in Supplementary Figure 1**b**. Panel **c** supplies an alternative rendering of the same data. The spectral gap as well as the expected edge modes can be clearly identified and are well aligned with the simulation in panel **a**, up to a rigid shift of the spectra. The COMSOL-simulated pressure fields of the edge resonant modes are shown in Supplementary Figure 1**d**.

Further simulations of the resonant spectra are reported in Supplementary Figure 2. These simulations are for finite crystals with periodic boundary conditions applied at the ends. Panel **a** corresponds to a metallic crystal with $t_{\rm in} = t_{\rm out} = 10mm$ and, as expected, the spectrum has no band gaps. Panel **b** corresponds to the SSH crystal with the geometry highlighted in Supplementary Figure 1**a** and has a gapped spectrum. Panel **c** corresponds to a metalized SSH chain, obtained by coupling the two systems considered in panels **a** and **b**. In this case the spectrum is again un-gapped. The reader should pay attention to the almost perfect chiral symmetry of the spectrum in all these three graphs.

SUPPLEMENTARY NOTE 2: THE LOCALIZER FOR CLASS BDI, ONE-DIMENSIONAL SYSTEMS

The localizer is a mathematical object that can be built from any set of $d+1$ Hermitian matrices. For a d-dimensional system, the most standard approach uses the d position observables, X_j for $j \in 1,\dots,d$, and the Hamiltonian, H, to form the generic localizer

$$\sum_{j=1}^{d}(X_j - x_j I)\otimes\Gamma_j + (H - EI)\otimes\Gamma_{d+1}.$$

Here, the Γ matrices form a non-trivial Clifford representation. There is no requirement that we use all of the position observables and we often want to modify the position observables to some degree. Most often, we find that the natural units for position may be too large or small, making the localizer unable to detect any interesting information. In that case, we can apply the localizer construction to $(\kappa X_1,\dots,\kappa X_d, H)$ and obtain

$$\sum_{j=1}^{d}(\kappa X_j - x_j I)\otimes\Gamma_j + (H - EI)\otimes\Gamma_{d+1}.$$

This function does not respect our initial units, so in physics applications we re-scale again the scalars x_j

* wc327@njit.edu; These two authors contributed equally
† awcerja@sandia.gov; These two authors contributed equally
‡ sc945@njit.edu
§ prodan@yu.edu
¶ loring@math.unm.edu
** cprodan@njit.edu

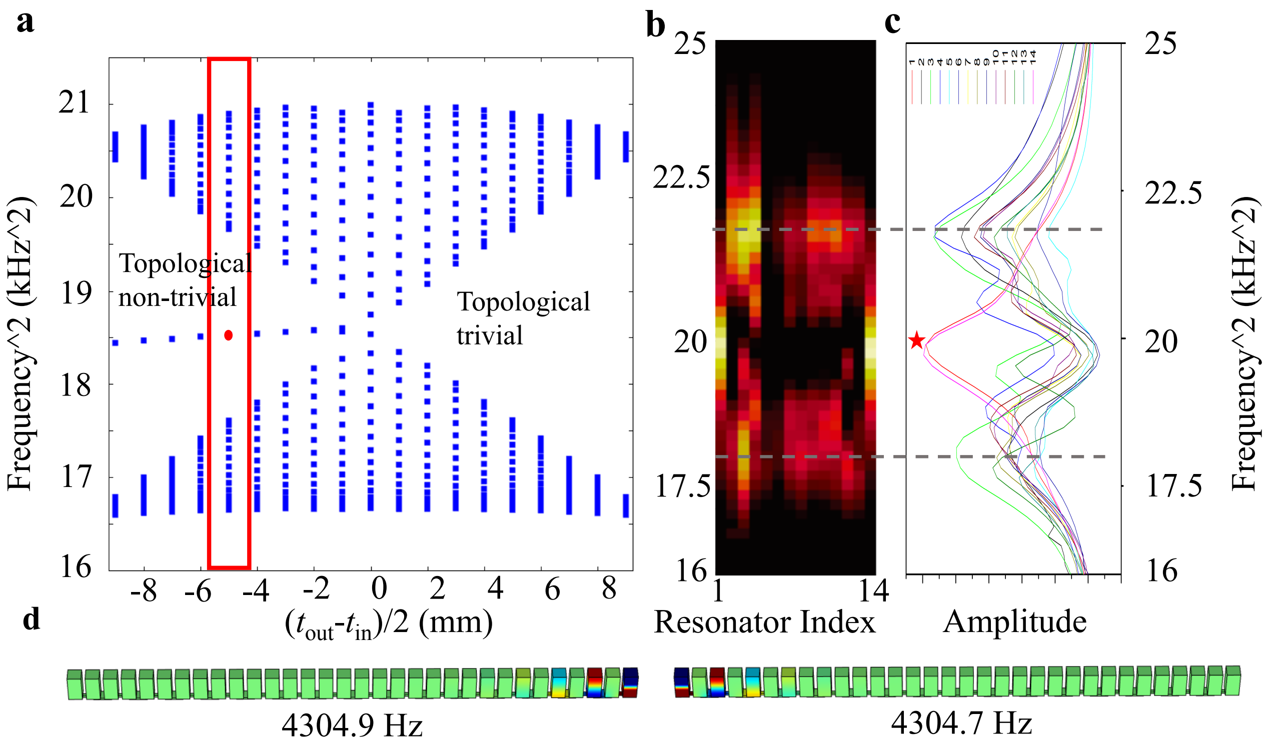

Supplementary Figure 1. **Experimental mapping of the SSH model resonant spectrum.** **a** COMSOL simulated SSH model resonant spectrum. The red vertical box indicates t_{in} = 15 mm and t_{out} = 5 mm, used in the experiments. **b** Experimentally measured local density of states, assembled from normalized microphone readings from the top of the block resonators. The bright dispersive modes indicates the bulk and edge modes. **c** Collapse on the frequency axis of the intensity plot in **b** . The spectral gap can be clearly identified and the edge modes show up in the gap marked as red asterisk. **d** Acoustic pressure field distribution for the edge modes marked as red dot in panel **a**

look at what we call the full spectral localizer in a d-dimensional system,

$$L_{(x_1,\ldots,x_d,E)}(X_1,\ldots,X_d,H) = \sum_{j=1}^{d} \kappa(X_j - x_j I)\otimes\Gamma_j + (H-EI)\otimes\Gamma_{d+1}. \quad (1)$$

Now we specialize to one-dimensional systems in the class BDI symmetry class. First, we make our choice of the Γ matrices. These need to anticommute with each other and each must be Hermitian and square to $-I$. The simplest choice here is $\Gamma_1 = \sigma_x$ and $\Gamma_2 = \sigma_y$, where $\sigma_{x,y}$ are the standard Pauli matrices, so we find

$$L_{(x,E)}(X,H) = \begin{bmatrix} 0 & \kappa(X-xI) - i(H-EI) \\ \kappa(X-xI) + i(H-EI) & 0 \end{bmatrix}.$$

In class BDI we have a grading operator specifying the chiral symmetry, with the assumptions $H\Pi = -\Pi H$ and $X\Pi = \Pi X$. The chiral operator Π is a unitary that squares to I. In most examples, Π and X are real matrices and we make those additional assumption, along with the standard assumption (for class BDI) that H is a real matrix. In this symmetry class and dimension, it is easier to work with a reduced matrix that is not always Hermitian,

$$\tilde{L}_{(x,E)}(X,H) = \kappa(X-xI)\Pi + H - iE\Pi.$$

This reduced localizer is Hermitian and real when $E = 0$ and that is where we shall focus. Indeed, it is

$$H_x = \tilde{L}_{(x,0)}(X,H) = \kappa(X-xI)\Pi + H.$$

The becomes the model Hamiltonian for the perturbed systems, see main text.

In this dimension and symmetry class, we call this the *localizer*, or reduced localizer if we need to distinguish from the full version. As expected, we can derive from this localizer the topological invariant and the measure of local topological protection.

Theorem 1. *Suppose Π is unitary with $\Pi^2 = 1$, and that X and H are Hermitian matrices with $H\Pi = -\Pi H$ and $X\Pi = \Pi X$. For all real scalars x and E*

$$\sigma_{\min}\left(\tilde{L}_{(x,E)}(X,H)\right) = \sigma_{\min}\left(L_{(x,E)}(X,H)\right),$$

where $\sigma_{\min}$ denotes smallest singular value.

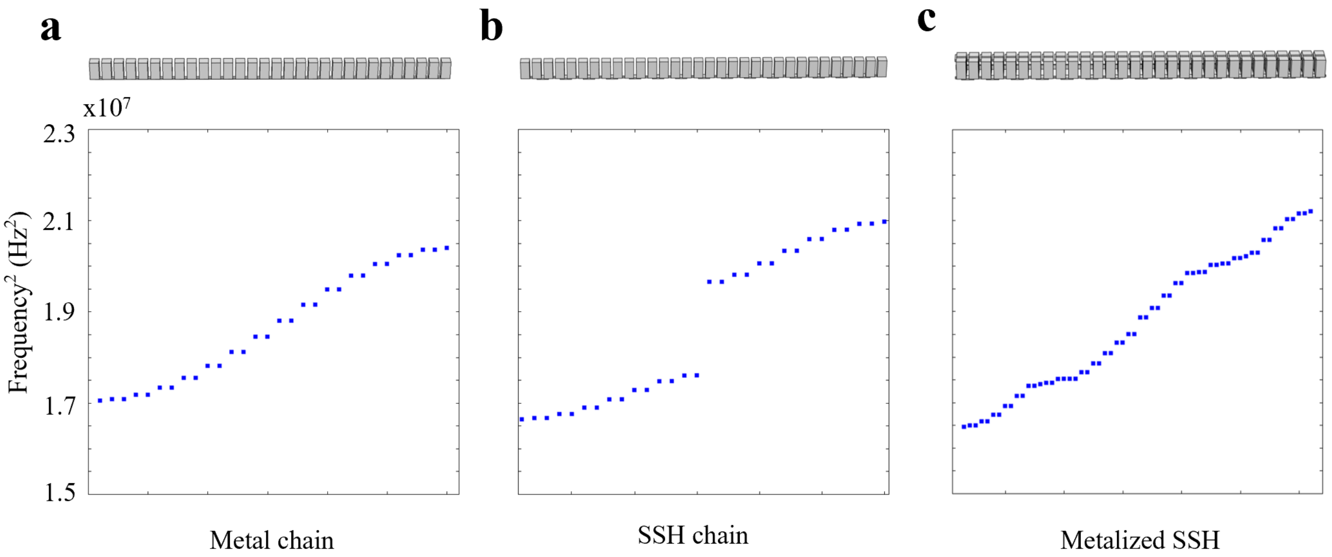

Supplementary Figure 2. **Dispersion of the acoustic modes a** Resonant spectrum of a periodic acoustic pattern. **b** Resonant spectrum of a SSH acoustic pattern. **c** Resonant spectrum of a metalized SSH acoustic pattern.

Proof. For any square matrix A, a standard math trick is to consider the larger matrix

$$M = \begin{bmatrix} 0 & A^\dagger \\ A & 0 \end{bmatrix}$$

that is always Hermitian. If we take a singular value decomposition $A = UDV^\dagger$, with D diagonal and positive and U and V unitary, we find

$$M = \begin{bmatrix} V & 0 \\ 0 & U \end{bmatrix} \begin{bmatrix} D & 0 \\ 0 & D \end{bmatrix} \begin{bmatrix} 0 & V \\ U & 0 \end{bmatrix}^\dagger .$$

This tells us that the spectrum of M consists of pairs of the form $\pm\lambda$ where λ is taken to be one of the singular values of A. If we apply this to $A = \kappa(X - xI) + i(H - EI)$ we discover

$$\sigma_{\min}\left(\kappa(X - xI) + i(H - EI)\right) = \sigma_{\min}\left(L_{(x,E)}(X, H)\right).$$

Since multiplying a matrix on one side by a unitary does not alter its singular values, we derive from this the equality

$$\sigma_{\min}\left(\kappa(X - xI)\Pi + i(H - EI)\Pi\right) = \sigma_{\min}\left(L_{(x,E)}(X, H)\right).$$

To finish the proof, we will show that $\kappa(X - xI)\Pi + i(H - EI)\Pi$ is unitarily equivalent to $\kappa(X - xI)\Pi + H - iE\Pi$. Let $\zeta = \frac{1}{2} - i\frac{1}{2}$ and define $Q = \zeta I + \bar{\zeta}\Pi$. This is unitary since

$$QQ^\dagger = \left(\zeta I + \bar{\zeta}\Pi\right)\left(\bar{\zeta} I + \zeta\Pi\right) = |\zeta|^2 I + \zeta^2\Pi + \bar{\zeta}^2\Pi + |\zeta|^2 I = I.$$

Since Π commutes with X and xI and EI, we see that Q commutes with all of these as well. We compute

$$\begin{aligned} Q\,(H\Pi)\,Q^\dagger &= \left(\zeta I + \bar{\zeta}\Pi\right)(H\Pi)\left(\bar{\zeta} I + \zeta\Pi\right) \\ &= |\zeta|^2 H\Pi + \zeta^2 H\Pi^2 + \bar{\zeta}^2\Pi H\Pi + |\zeta|^2\Pi H\Pi^2 \\ &= |\zeta|^2 H\Pi + \zeta^2 H + \bar{\zeta}^2\Pi H\Pi + |\zeta|^2\Pi H \\ &= |\zeta|^2 H\Pi + \zeta^2 H - \bar{\zeta}^2 H - |\zeta|^2 H\Pi \\ &= -iH, \end{aligned}$$

which means

$$Q\,(\kappa X\Pi + iH\Pi - (\kappa x + iE)\Pi)\,Q^\dagger = \kappa X\Pi + H - \kappa x I - iE\Pi.$$

□

Thus, in our situation, the reduced localizer, $\tilde{L}$, remains real-symmetric at $E = 0$. Of course, it is only at $E = 0$ that we can extract topological information, as the usual local index in only defined at $E = 0$ and for those x where $L_{(x,0)}(X, H)$ is invertible. Then we look up the relevant index formula in [1, § 4.2] and find the index is then

$$\nu(x) = \frac{1}{2}\operatorname{sig}\left(\tilde{L}_{(x,0)}(X, H)\right). \qquad (2)$$

The index can also be computed using the class AIII formula, which uses the signature of $(X - xI)\Pi + iH\Pi$. This fails to remain a real matrix so is not preferred.

SUPPLEMENTARY NOTE 3: ALTERING THE RE-CENTERED POSITION OPERATOR

In this study, it is necessary to attenuate the growth of the $\kappa(X_j - x_j I) \otimes \Gamma_j$ terms in Eq. (4) in the main text so

that each resonator's fundamental axial mode maintains a similar modal profile (to enable resonator couplings) and a frequency that is separated from the remainder of the spectrum. Thus, instead of using $\kappa(X_j - x_j I)$ we are using

$$\kappa \tanh(\alpha^{-1}(X_j - x_j I))$$

which is applying to $X_j - x_j I$ a function that is approximately linear near at zero. Since the localizer is, in fact, local, this makes only a minor difference in the mathematical analysis [2, 3].

If, after selecting $(x_1, \cdots, x_d)$, we truncate the system, the eigenvalues near 0 of the localizer are not much effected if we truncate the systems in space, removing parts of the system far from $(x_1, \cdots, x_d)$. The best bounds on this can be derived from [3, §VI]. We can then apply Weyl's inequality on Hermitian matrices to the spectrum of the locacalizer. In this way, we see that when analyzing the spectrum of the locazlizer close to 0 we are justified in using the approximation

$$\kappa \tanh(\alpha^{-1}(X_j - x_j I)) \approx (\kappa\alpha^{-1})(X_j - x_j I)).$$

SUPPLEMENTARY NOTE 4: SECONDARY INDEX OF THE SPECTRAL LOCALIZER

The local gap gives an indication of the strength of the protection given by the associated topological index. By the local gap, we mean

$$\mu_{(x,0)} = \sigma_{\min}\left(\tilde{L}_{(x,0)}(X, H)\right). \tag{3}$$

The relevance is that one can prove [1, § 7] that it takes a perturbation of H whose size (l_2 matrix norm) is at least equal to this local gap to make the local index change. Thus, the positions (different choices of x), where the local index changes, are more protected when the local gaps on either side are large. The local index can only change when the local gap goes to zero, and these points correspond to localized states [1, 3].

In the case of systems with a well-defined bulk gap, it is now established that the local index equals a more traditional bulk index [4, 5]. This was anticipated when the local index was defined. In this work we are pushing the localizer index into uncharted waters. What we find is that there is substantial additional information in the localizer's spectrum beyond the gap at the center of its spectrum. This was seen previously in the case of semimetals by Schulz-Baldes and Stoiber [6].

In our case, we observe one eigenvalue moving (as we move x) from close to the center of the spectrum to well above, and another from close to the localizer's spectrum's center to well below. The proofs about topological protection in [1] only depend on estimates on spectral variation for Hermitian matrices so apply equally well to gaps in the localizer spectrum that are not at zero. That is, we can define an secondary index

$$\nu_{\xi}(x) = \frac{1}{2}\operatorname{sig}\left(\tilde{L}_{(x,0)}(X, H) - \xi I\right). \tag{4}$$

whose strength can be inferred from

$$\mu_{\xi,(x,0)} = \sigma_{\min}\left(\tilde{L}_{(x,0)}(X, H) - \xi I\right). \tag{5}$$

We are looking for ξ where there are two values x_1 and x_2 for x that lead to large secondary gaps and distinct values of the secondary index. As long as $|\xi|$ is not too large we will still be able to conclude there is a state with some localization and some topological protection.

Let us now be more precise on how small we require $|\xi|$ so that the secondary index $\mu_{\xi,(x,0)}$ will be still meaningful. A change in this index will mean that even after adding disorder there will be a nearby x where

$$\xi \in \sigma\left(\tilde{L}_{(x,0)}(X, H)\right)$$

As we show above, this is equivalent to

$$\xi \in \sigma\left(L_{(x,0)}(X, H)\right)$$

and so we conclude

$$\sigma_{\min}(L_{(x,0)}(X, H)) \leq \xi.$$

From Proposions II.1 and II.4 of [3] we know there is a state $\boldsymbol{\psi}$ with the following bounds on how it is localized at position x and energy 0:

$$\sqrt{\kappa^2 \Delta^2_{\psi} X + \Delta^2_{\psi} H + \kappa^2 \left(\mathrm{E}_{\psi}[X] - x\right)^2 + \mathrm{E}_{\psi}[H]^2} \leq \sqrt{\xi^2 + \kappa \,\|[H, X]\|}$$

Here we are using $\mathrm{E}_{\psi}[O]$ and $\Delta^2_{\psi} O$ to indicate expectation and square of variance of an observable given this state. When $\xi = 0$ we are getting localization error that might be as bad as $\sqrt{\kappa \,\|[H, X]\|}$, which is as we expect due to the limitations imposed by various uncertainly principals. If we restrict ourselves to secondary indices with

$$\xi \leq \sqrt{3\kappa \,\|[H, X]\|} \tag{6}$$

then the proven bounds on localization errors will only double.

SUPPLEMENTARY NOTE 5: BULK-INTERFACE CORRESPONDENCE PRINCIPLE

A material or metamaterial is usually declared topological if it has a well defined bulk-interface correspondence principle. The latter refers to ones ability to predict the interface physics by examining topological data faraway from the interface, hence, in the bulk of the two

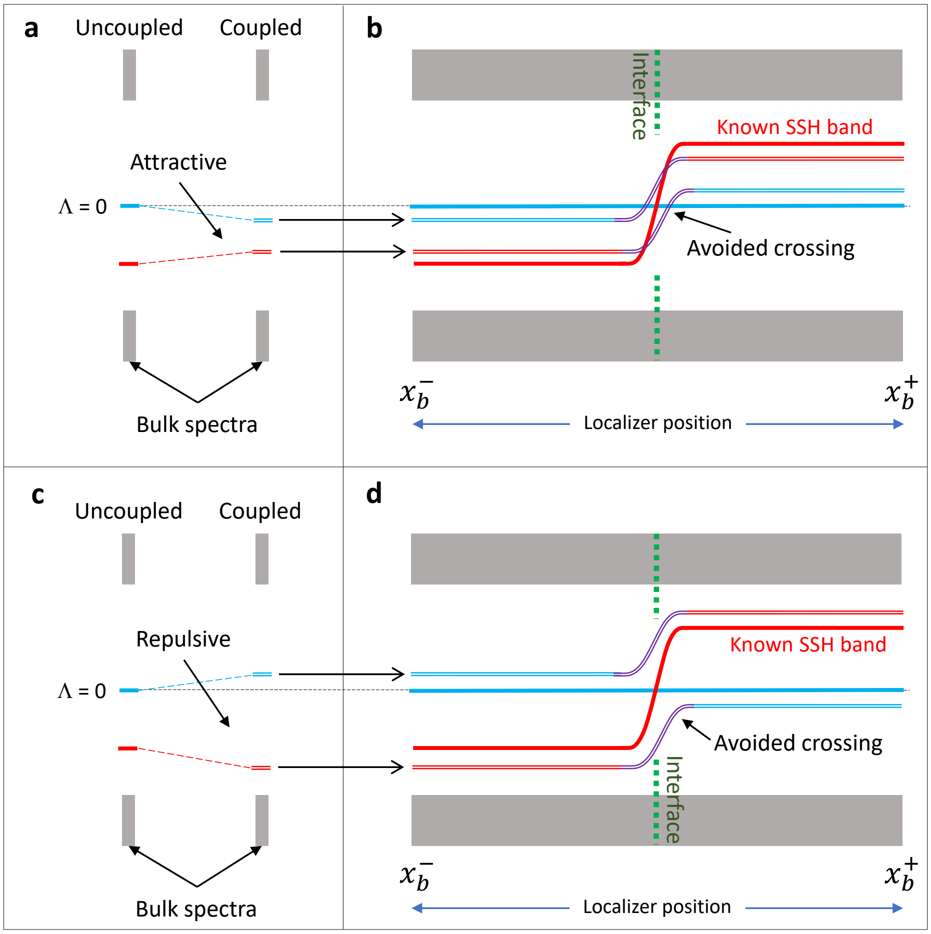

Supplementary Figure 3. **Interface physics derived from the bulk spectral data of the spectral localizer.** **a** The attractive scenario, leading to a spectral flow that induces a change by two units of the spectral localizer index at the interface. **b** The repulsive scenario, leaving the spectral index constant as one traverses the interface.

interfaced materials. The main claim of our work is that this is indeed the case for the metallic system we investigate here, for which the bulk topological data can be read off from the spectrum of the spectral localizer, as it was done in Fig. 4 of the main text. In this section, we elaborate on this statement and explain the spectral flow seen in Fig. 4**a** of our main text.

We start by observing that our uncoupled metallic chain from the SSH class can be seen as a critical phase between two topological insulating SSH phases. For the latter, we already know that, in the bulk, the spectral localizer $\tilde{L}_{x,0}(X, H)$ displays a gapped spectrum and one eigenvalue close to $E = 0$, either above or below, depending on the SSH phase. Since the metal SSH is in between the two distinct insulating SSH phases, we can safely conclude that the spectral localizer for our decoupled metallic chain will display a gapped bulk spectrum and one eigenvalue pinned exactly at $E = 0$. Supplementary Figure 3 illustrates the typical bulk spectrum of $\tilde{L}_{x,0}(X, H)$, when the metallic and the insulating chains are decoupled. Upon coupling, the eigenvalues close to $E = 0$ can either attract or repel and, in both cases, the eigenvalue originally pinned at zero is robustly moved away from $E = 0$. The direction in which this eigenvalue moves is encoded in the index $\nu(x_{\rm bulk})$. Indeed, for the attractive case illustrated in Supplementary Figure 3**a**, we have $\nu(x) = -1$, while for the repulsive case $\nu(x) = 0$. Although we have not observed the latter case, both and many more scenarios are in principle possible.

Now, in the presence of an interface, one can collect the spectral data of the localizer far from the interface and edges, for both left and right bulk regions, say at $x_{\rm b}^{\pm}$. With this data at hand, one can easily predict the spectral flow of the localizer $\tilde{L}_{x,0}(X, H)$ as x is varied across the interface. Indeed, starting from the uncoupled regime and using the known behavior of the spectral localizer for the insulating SSH phases, one draws the two crossing bands bands seen in Supplementary Figure 3**b**,**d** (focus on the solid lines). Then, using the bulk spectral data together with the standard avoiding crossing principle, one can easily derive the spectral flow of the localizer in the coupled regime. This is illustrated by the doubled-lines in Supplementary Figure 3**b**,**d**, for the attractive and repulsive cases, respectively. Clearly, there is a strong re-

semblance between the spectral flow from Supplementary Figure 3**b** and the spectral flow reported in Fig. 4 of our main text. Hence, our qualitative analysis explains the spectral flows observed in the COMSOL simulation and experiment, whose origin can be now pinpointed to the attraction between the relevant eigenvalues of the spectral localizer.

There is a stark difference between the attractive and repulsive cases reported in Supplementary Figure 3. In the attractive case, the local spectral gap $\mu_{(x,0)}$ closes twice and, as already mentioned, each closing of $\mu_{(x,0)}$ implies the existence of a localized resonant mode. Thus, from the spectral flow from Supplementary Figure 3**b**, one can safely conclude the existence of two resonant modes localized at the interface, in complete agreement with the numerical and experimental observations discussed in the main text. In contradistinction, from the spectral flow Supplementary Figure 3**d** where repulsive case is shown, one will conclude the absence of such modes. Stepping back for a second, one realizes that, qualitatively, the spectral flows reported in Supplementary Figure 3 can be derived entirely from the local bulk indices $\nu(x_b^{\pm})$ and, based on the possible resulting spectral flows, the following principle emerges: There is a stark difference between the attractive and repulsive cases reported in Supplementary Figure 3. In the attractive case, the local spectral gap $\mu_{(x,0)}$ closes twice and, as already mentioned, each closing of $\mu_{(x,0)}$ implies the existence of a localized resonant mode. Thus, from the spectral flow from Supplementary Figure 3**b**, one can safely conclude the existence of two resonant modes localized at the interface, in complete agreement with the numerical and experimental observations discussed in the main text. In contradistinction, from the spectral flow Supplementary Figure 3**d** where repulsive case is shown, one will conclude the absence of such modes. Stepping back for a second, one realizes that, qualitatively, the spectral flows reported in Supplementary Figure 3 can be derived entirely from the local bulk indices $\nu(x_b^{\pm})$ and, based on the possible resulting spectral flows, the following principle emerges: There is a stark difference between the attractive and repulsive cases reported in Supplementary Figure 3. In the attractive case, the local spectral gap $\mu_{(x,0)}$ closes twice and, as already mentioned, each closing of $\mu_{(x,0)}$ implies the existence of a localized resonant mode. Thus, from the spectral flow from Supplementary Figure 3**b**, one can safely conclude the existence of two resonant modes localized at the interface, in complete agreement with the numerical and experimental observations discussed in the main text. In contradistinction, from the spectral flow Supplementary Figure 3**d** where repulsive case is shown, one will conclude the absence of such modes. Stepping back for a second, one realizes that, qualitatively, the spectral flows reported in Supplementary Figure 3 can be derived entirely from the local bulk indices $\nu(x_b^{\pm})$ and, based on the possible resulting spectral flows, the following principle emerges: There is a stark difference between the attractive and repulsive cases reported in Supplementary Figure 3. In the attractive case, the local spectral gap $\mu_{(x,0)}$ closes twice and, as already mentioned, each closing of $\mu_{(x,0)}$ implies the existence of a localized resonant mode. Thus, from the spectral flow from Supplementary Figure 3**b**, one can safely conclude the existence of two resonant modes localized at the interface, in complete agreement with the numerical and experimental observations discussed in the main text. In contradistinction, from the spectral flow There is a stark difference between the attractive and repulsive cases reported in Supplementary Figure 3. In the attractive case, the local spectral gap $\mu_{(x,0)}$ closes twice and, as already mentioned, each closing of $\mu_{(x,0)}$ implies the existence of a localized resonant mode. Thus, from the spectral flow from Supplementary Figure 3**b**, one can safely conclude the existence of two resonant modes localized at the interface, in complete agreement with the numerical and experimental observations discussed in the main text. In contradistinction, from the spectral flow There is a stark difference between the attractive and repulsive cases reported in Supplementary Figure 3. In the attractive case, the local spectral gap $\mu_{(x,0)}$ closes twice and, as already mentioned, each closing of $\mu_{(x,0)}$ implies the existence of a localized resonant mode. Thus, from the spectral flow from Supplementary Figure 3**b**, one can safely conclude the existence of two resonant modes localized at the interface, in complete agreement with the numerical and experimental observations discussed in the main text. In contradistinction, from the spectral flow Supplementary Figure 3**d** where repulsive case is shown, one will conclude the absence of such modes. Stepping back for a second, one realizes that, qualitatively, the spectral flows reported in There is a stark difference between the attractive and repulsive cases reported in Supplementary Figure 3. In the attractive case, the local spectral gap $\mu_{(x,0)}$ closes twice and, as already mentioned, each closing of $\mu_{(x,0)}$ implies the existence of a localized resonant mode. Thus, from the spectral flow from Supplementary Figure 3**b**, one can safely conclude the existence of two resonant modes localized at the interface, in complete agreement with the numerical and experimental observations discussed in the main text. In contradistinction, from the spectral flow There is a stark difference between the attractive and repulsive cases reported in Supplementary Figure 3. In the attractive case, the local spectral gap $\mu_{(x,0)}$ closes twice and, as already mentioned, each closing of $\mu_{(x,0)}$ implies the existence of a localized resonant mode. Thus, from the spectral flow from Supplementary Figure 3**b**, one can safely conclude the existence of two resonant modes localized at the interface, in complete agreement with the numerical and experimental observations discussed in the main text. In contradistinction, from the spectral flow Supplementary Figure 3**d** where repulsive case is shown, one will conclude the absence of such modes. Stepping back for a second, one realizes that, qualitatively, the spectral flows Supplementary Figure 3 can be derived entirely from the local bulk in-

dices $\nu(x_b^{\pm})$ and, based on the possible resulting spectral flows, the following principle emerges:Supplementary Figure 3**d** where repulsive case is shown, one will conclude the absence of such modes. Stepping back for a second, one realizes that, qualitatively, the spectral flows reported in Supplementary Figure 3 can be derived entirely from the local bulk indices $\nu(x_b^{\pm})$ and, based on the possible resulting spectral flows, the following principle emerges:Supplementary Figure 3 can be derived entirely from the local bulk indices $\nu(x_b^{\pm})$ and, based on the possible resulting spectral flows, the following principle emerges: 3**d** where repulsive case is shown, one will conclude the absence of such modes. Stepping back for a second, one realizes that, qualitatively, the spectral flows reported in Supplementary Figure 3 can be derived entirely from the local bulk indices $\nu(x_b^{\pm})$ and, based on the possible resulting spectral flows, the following principle emerges: 3**d** where repulsive case is shown, one will conclude the absence of such modes. Stepping back for a second, one realizes that, qualitatively, the spectral flows reported in Supplementary Figure 3 can be derived entirely from the local bulk indices $\nu(x_b^{\pm})$ and, based on the possible resulting spectral flows, the following principle emerges:

$$\boxed{\#\ \text{localized interface modes} \geq |\nu(x_b^+) - \nu(x_b^-)|.} \tag{7}$$

The fact that we have an inequality instead of an equality comes from the fact that the spectral flow may not always be monotone as in Supplementary Figure 3**b,d**, but can display oscillations, depending on how complicated the interface potential is.

Finally, this argument gives us the means to predict what will happen if a repulsive case is interfaced with attractive case, in which case we have $|\nu(x_b^+) - \nu(x_b^-)| = 1$, hence the expectation is that at least one localized interface mode shows up. This scenario occurs at the edges of our samples, as it will be revealed next.

SUPPLEMENTARY NOTE 6: REALIZATION IN TIGHT-BINDING

Our acoustic metamaterial can be approximated using a tight binding model, whose topology can be tuned by changing the relative strengths of the intra- and inter-unit cell couplings, t_{in} and t_{out}, respectively. The lattice Hamiltonian for the two coupled chains can be written as

$$\begin{aligned} H = &- t_{\text{in}} \sum_j a^\dagger_{j,\text{SSH}} b_{j,\text{SSH}} - t_{\text{out}} \sum_j b^\dagger_{j,\text{SSH}} a_{j+1,\text{SSH}} \\ &- t_{\text{M}} \sum_j (b^\dagger_{j,\text{M}} a_{j,\text{M}} + a^\dagger_{j,\text{M}} b_{j+1,\text{M}}) \\ &- t_{\text{c}} \sum_j (a^\dagger_{j,\text{SSH}} b_{j,\text{M}} + b^\dagger_{j,\text{SSH}} a_{j,\text{M}}) + \text{c.c.} \end{aligned} \tag{8}$$

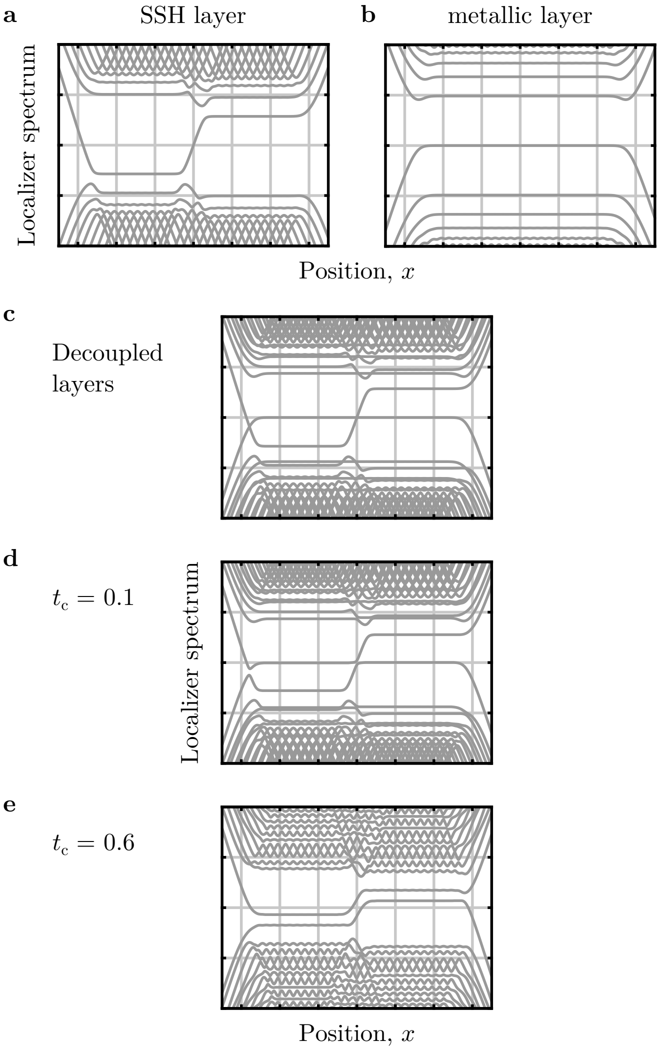

Supplementary Figure 4. **Evolution of the localizer's spectrum as the inter-layer coupling is increased. a** Localizer spectrum at $E = 0$ for the SSH layer in isolation, with $t_{\text{in}} = 0.4$ and $t_{\text{out}} = 1$. **b** Similar, except for the metallic layer in isolation, with $t_{\text{M}} = 1$. **c**, **d**, **e** Localizer spectrum at $E = 0$ for the two-layer system, with $t_{\text{in}} = 0.4$, $t_{\text{out}} = 1$, $t_{\text{M}} = 0.8$, and $t_c = 0$, 0.1, and 0.6.

Here, $a_{j,\text{SSH}}$, $b_{j,\text{SSH}}$ ($a^\dagger_{j,\text{SSH}}$, $b^\dagger_{j,\text{SSH}}$) are the annihilation (creation) operators for the left and right sites of the jth unit cell in the bipartite SSH chain. Similarly, $b_{j,\text{M}}$, $a_{j,\text{M}}$ ($b^\dagger_{j,\text{M}}$, $a^\dagger_{j,\text{M}}$) are the annihilation (creation) operators for the left and right sites of the jth unit cell in the uniform gapless chain, where the ordering of a and b to left and right has been switched to emphasize the system's chiral symmetry. The coupling strength within the uniform layer is t_{M}, and the two layers are coupled together with strength t_c.

The localizer spectra at $E = 0$, $\tilde{L}_{(x,0)}(X, H)$, for the SSH and metallic layers separately are shown in Supplementary Figure 4**a**,**b**. Thus, the localizer spectrum for the two layers together, but decoupled ($t_c = 0$), can be seen to the the superposition of these two spectra, Supple-

mentary Figure 4**c**. Then, as the inter-layer coupling is increased, we numerically observe the attraction within the localizer's spectrum, Supplementary Figure 4**d,e**.

SUPPLEMENTARY REFERENCES

[1] T. A. Loring, Annals of Physics **356**, 383 (2015).

[2] T. A. Loring, Preprint at http://arxiv.org/abs/1907.11791 (2019).

[3] A. Cerjan, T. A. Loring, and F. Vides, J. Math. Phys. **64**, 023501 (2023).

[4] T. A. Loring and H. Schulz-Baldes, New York J. Math. **23**, 1111 (2017).

[5] T. A. Loring and H. Schulz-Baldes, J. Noncommut. Geom. **14**, 1 (2020).

[6] H. Schulz-Baldes and T. Stoiber, EPL **136**, 27001 (2021).
